# Quantitative Assessment of Finger Motor Impairment in Multiple Sclerosis

**DOI:** 10.1371/journal.pone.0065225

**Published:** 2013-05-31

**Authors:** Laura Bonzano, Maria Pia Sormani, Andrea Tacchino, Lucia Abate, Caterina Lapucci, Giovanni Luigi Mancardi, Antonio Uccelli, Marco Bove

**Affiliations:** 1 Department of Neuroscience, Rehabilitation, Ophthalmology, Genetics, Maternal and Child Health, University of Genoa, Genoa, Italy; 2 Department of Health Sciences, Biostatistics Unit, University of Genoa, Genoa, Italy; 3 Department of Experimental Medicine, Section of Human Physiology and Centro Polifunzionale di Scienze Motorie, University of Genoa, Genoa, Italy; University Medical Center Groningen UMCG, The Netherlands

## Abstract

**Objective:**

To address the disability impact on fine hand motor functions in patients with Multiple Sclerosis (MS) by quantitatively measuring finger opposition movements, with the aim of providing a new “score” integrating current methods for disability assessment.

**Methods:**

40 MS patients (Expanded Disability Status Scale (EDSS): 0–7) and 80 healthy controls (HC) performed a repetitive finger-to-thumb opposition sequence with their dominant hand at spontaneous and maximal velocity, and uni- and bi-manually metronome-paced. A sensor-engineered glove was used to measure finger motor performance. Twenty-seven HC were tested twice, one month apart, to assess test-retest reliability.

**Results:**

The motor parameters showed a good reproducibility in HC and demonstrated significantly worse performance in MS patients with respect to HC. A multivariate model revealed that rate of movement in the spontaneous velocity condition and inter-hand interval (IHI), indicating bimanual coordination, contributed independently to differentiate the two groups. A finger motor impairment score based on these two parameters was able to discriminate HC from MS patients with very low EDSS scores (p<0.001): a significant difference was already evident for patients with EDSS = 0. Further, in the MS group, some motor performance parameters correlated with the clinical scores. In particular, significant correlations were found between IHI and EDSS (r = 0.56; p<0.0001), MS Functional Composite (r = −0.40; p = 0.01), Paced Auditory Serial Addition (r = −0.38; p = 0.02). No motor performance parameter correlated with Timed 25-Foot Walk.

**Conclusions:**

A simple, quantitative, objective method measuring finger motor performance could be used to define a score discriminating healthy controls and MS patients, even with very low disability. This sensitivity might be of crucial importance for monitoring the disease course and the treatment effects in early MS patients, when changes in the EDSS are small or absent.

## Introduction

Strategies to improve the disability assessment in Multiple Sclerosis (MS) have been the focus of discussion of the International Conference on Disability Outcomes in MS (Washington DC, May 2011) [1]. In fact, the Expanded Disability Status Scale (EDSS) [2] is commonly used as primary outcome measure in MS clinical trials, but its limitations are well known [3], [4]. Particularly, it is heavily weighted toward ambulation and fails to assess cognition and upper extremity function. A first attempt to overcome the weakness of the EDSS is represented by the MS Functional Composite (MSFC) [5], including the 9-Hole Peg Test (9-HPT) to measure arm/hand function.

Here, we propose to assess the disability impact on fine hand motor functions in MS by quantitatively measuring finger opposition movements, with the goal to re-balance the motor disability assessment enhancing upper limb evaluation. Indeed, finger movements are crucial in daily-life activities, e.g. writing, typing or more generally manipulating objects. So far, very few studies based on small cohorts of patients have been carried out [6], [7], [8]. Recently, we demonstrated that patients with MS show general motor slowing and impaired bimanual coordination [9], [10], [11]. Therefore, in this study we could expect to find out finger motor impairments in a large group of MS patients with respect to a group of healthy subjects, even in those patients considered with very low disability as mainly evaluated on the lower limb.

Also, taking into account the relationships of the new measures with known clinical scales, we could hypothesize that the proposed methodology was able to give information specific for the upper limb, providing a new “score” integrating current methods for evaluating disability. In this line, we could hypothesize to observe significant correlations only with some variables (e.g., not strictly related to the lower limb) and we could also generally expect low correlation coefficients, because the proposed methodology was thought to test domains (e.g., the ability to perform fine distal movements in their different aspects including spatio-temporal accuracy and bimanual coordination) not fully accounted for by the available clinical scales.

On these bases, aims of the present work were: (a) assessing the reproducibility of repeated measurements of finger motor performance; (b) investigating in a large sample of subjects the ability of measures related to repetitive finger opposition movements to discriminate MS from healthy subjects; (c) identifying the finger motor performance parameters able to better describe the patient's finger motor impairment; (d) evaluating the relationship between finger motor performance and the commonly used clinical scales (EDSS and MSFC).

## Materials and Methods

### Ethics statement

Informed written consent was obtained from all subjects and the study was approved by the ethics committee of the University of Genoa.

### Subjects

We enrolled 40 patients with MS (MS group: 37 relapsing-remitting and 3 secondary-progressive MS patients, 27 females (67%) and 13 males (33%), mean age±standard deviation (SD): 42±9 years) and 80 age- and sex-matched healthy controls (HC group: 49 females (61%) and 31 males (39%), mean age±SD: 41±10 years).

Patients with MS were in a stable phase of the disease, without relapses or worsening greater than one point on the EDSS in the last three months. EDSS ranged from 0 to 7 (median = 2) and disease duration from 8 months to 42 years (median = 11 years). We also acquired the MSFC score (mean MSFC±SD = −0.71±1.4). Moreover, fatigue was evaluated by the Modified Fatigue Impact Scale (mean MFIS±SD = 26.8±13.7), a modified form of the Fatigue Impact Scale [12].

None of the subjects included in the HC group presented with a history of neurological or psychiatric disorders or use of psychoactive drugs.

All the subjects were right-handed according to a modified Italian-translated Edinburgh Handedness Inventory [13] and naive to the specific purpose of this study.

### Experimental procedure

Subjects were asked to perform repetitive finger opposition movements of thumb to index, medium, ring and little fingers, self-paced or paced with an external cue. In details, they had to perform the finger motor sequence with the dominant hand (i.e., right for all the included subjects) at their spontaneous (SV condition) and maximal (MV condition) velocity. Then, the finger motor sequence had to be paced with a metronome tone set at a rate of 2 Hz (2 Hz condition), that is slightly lower than the spontaneous speed of healthy subjects performing the same finger motor task [14]. The 2 Hz condition was repeated with both hands simultaneously (2 Hz_bim condition). An eyes-closed paradigm was chosen to exclude possible confounding effects attributable to the integration of acoustic and visual information and to prevent patients from compensating for possible sensorimotor impairments by visual inspection.

To measure finger opposition movements, subjects wore a sensor-engineered glove on both hands (GAS, ETT s.r.l., Italy). In particular, from our previous experience, the proposed technique has been demonstrated to be reproducible across trials and by different examiners, brief and easy to administer also by non-doctoral personnel and widely acceptable to patients, reflecting the majority of the characteristics of an optimal clinical outcome measure [5].

Data were acquired at 1 KHz by means of a data acquisition board (USB-1208FS, Measurement Computing, USA). An ad hoc software tool generated the acoustic pacing signal, which was delivered by the system and listened by the subjects through isolation headphones, and recorded the occurrence of each tone and of each finger touch in the motor sequence.

The testing session included four randomly presented 60-s trials (one per condition). Before recording, all subjects practiced the task at their own spontaneous pace; training ended generally within 2 min when they were able to perform the finger motor sequence without errors. Thus, no training effect was present in this evaluation protocol, also considering that the proposed finger motor sequence was rather simple because the fingers had to be touched in the correct order (there was no sequence to learn). The evaluated parameters were an average on a large number of touches (e.g., 30 sequences in the metronome-paced trials, equivalent to 120 touches). The entire protocol lasted about 10 min, including 1-min rest periods between two consequent trials.

Twenty-seven HC (mean age = 42 years, range 26–69 years) were tested twice, one month apart, to assess the variation in measurements taken under the same conditions and define the motor performance parameters showing good repeatability (i.e., test-retest reliability).

### Behavioral data analysis

To describe the different aspects of the investigated motor task, the behavioral data collected with the glove system were processed with customized software, and different parameters were defined for the specific analysis. Touch Duration (TD) was computed as the contact time between the thumb and another finger, while Inter Tapping Interval (ITI) was defined as the time interval between the end of a thumb-to-finger contact and the beginning of the subsequent contact in the finger motor sequence. Hence, movement rate (RATE) could be computed as 1/(TD+ITI). For the bimanual trial (2 Hz_bim), bimanual coordination was assessed by means of the Inter Hand Interval (IHI), calculated as the absolute time difference between the touch onset occurring in the left hand and the corresponding touch in the right hand: the larger the IHI value, the more severe the impairment in bimanual coordination [9].

### Statistical data analysis

The test-retest reliability of the different motor performance parameters was assessed by the Intraclass Correlation Coefficient (ICC), quantifying the absolute agreement for both a single measurement and the average of two repetitions. The Smallest Real Difference (SRD), quantifying the changes that are detectable in longitudinal evaluations, was calculated for each parameter.

Mann-Whitney U test was used to assess differences in the parameters obtained by the glove system between the two groups (HC and MS).

The motor performance parameters found to be significantly different between the two groups were then entered in a step-wise logistic regression model adjusting for age effects, to find out the parameters independently able to discriminate patients with MS from healthy subjects. Before the inclusion, IHI was log-transformed to adjust for its skewed distributions.

A ROC curve analysis and the area under the curve (AUC) were used to assess the discriminating ability of the final model (concurrent criterion validity). The area under the ROC curve measures the accuracy of the combinations of variables included in the final model in discriminating HC from MS patients: an area of 100% represents a perfect discrimination, while an area of 50% represents a worthless model. Also, a leave-one-out cross-validation procedure was used to assess the accuracy of the final model in discerning healthy subjects and patients with MS, in order to mitigate the overfitting effect related to testing the performance of a model on the same dataset used to set its parameters.

The weighted combination of motor performance parameters (“Finger Motor Impairment score”) generated by the logistic model was compared across groups with different EDSS values using an analysis of variance (ANOVA) model.

Partial correlations (adjusted for age) were assessed on ranked variables, evaluating the relationships between the finger motor performance parameters and the clinical scales (EDSS, MSFC, and MFIS) to have an estimate of the concurrent criterion validity of the glove parameters.

## Results

### Test-retest reliability of repeated measurements of finger motor performance

Figure? 1 shows the hand motor performance parameters in the two repetitions (trial 2 vs. trial 1) performed one-month apart by a group of healthy subjects, with respect to the identity (i.e., the bisector). The SRD (%) ranged between 20% and 35% (Table? 1); this finding has to be considered in case of longitudinal studies, in fact glove parameters should exceed these values to indicate clinically significant changes in patients. The test-retest reliability resulted to be very high for RATE in the MV condition and IHI in the 2 Hz_bim condition, with an ICC ranging from 71% to 75% for a single measurement and from 83% to 86% for the average of two measurements. The reproducibility of TD (2 Hz), RATE (SV) and ITI (2 Hz) was quite good when based on two repetitions (69%, 63%, 55%, respectively).

**Figure 1:**
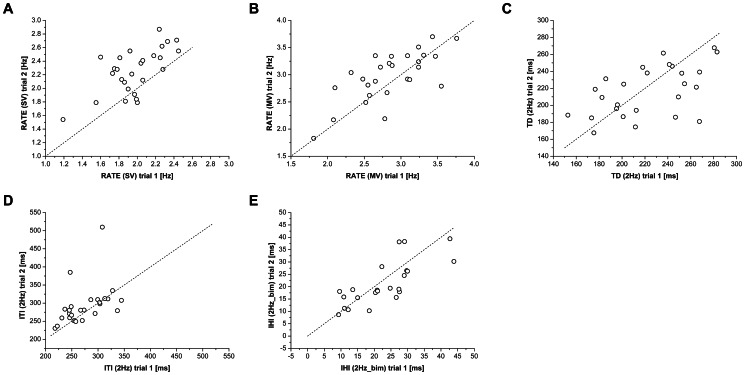
Test-retest reliability of the motor performance parameters. **(A-E)** Motor performance parameters in the two sessions performed one-month apart in a group of healthy controls (trial 2 vs. trial 1), with respect to the identity (i.e., the dashed line represents the bisector).

**Table 1:** Mean values for each session with standard deviations (SD), absolute and percentage smallest real difference (SRD) and intraclass correlation coefficient (ICC) for each motor performance parameter.

	Mean±SD				ICC (absolute agreement)	
Parameter (condition) [unit]	Session 1	Session 2	SRD	SRD (%)	Single	Average
RATE (SV) [Hz]	1.97±0.29	2.26±0.33	0.51	24	0.46	0.63
RATE (MV) [Hz]	2.86±0.48	2.98±0.45	0.69	24	0.71	0.83
TD (2 Hz) [ms]	222.8±37.0	217.2±29.4	63.8	29	0.53	0.69
ITI (2 Hz) [ms]	274.9±37.0	293.2±55.1	100.1	35	0.39	0.55
IHI (2 Hz_bim) (log-transformed)	3.03±0.45	2.97±0.42	0.61	20	0.75	0.86

SV = Spontaneous Velocity condition. MV = Maximal Velocity condition. 2 Hz = metronome condition (tone set at a rate of 2 Hz). 2 Hz_bim = metronome condition with both hands simultaneously. RATE = movement speed. TD = Touch Duration. ITI = Inter Tapping Interval. IHI = Inter Hand Interval. SD = Standard Deviation. SRD = Smallest Real Difference. ICC = Intraclass Correlation Coefficient.

### Comparison of motor performance parameters between MS patients and healthy subjects

The mean values of the motor performance parameters collected by the glove system were compared between the two groups (HC and MS) (Figure? 2).

**Figure 2:**
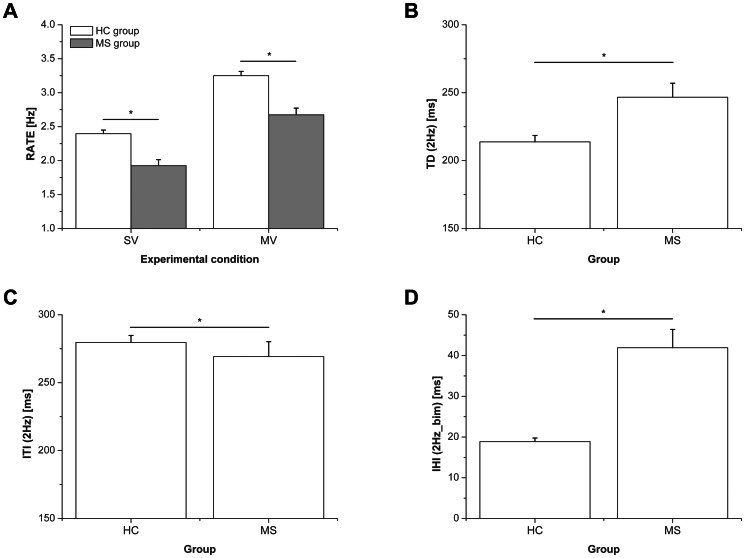
Comparison of the motor performance parameters between the two groups (HC and MS). (A) RATE in the spontaneous and maximal velocity conditions, (B) Touch Duration in the 2 Hz condition, (C) Inter Tapping Interval in the 2 Hz condition, (D) Inter Hand Interval in the 2 Hz_bim condition. * indicates statistically significant difference between the two groups (HC and MS). Error bars indicate standard error of the mean.

When evaluating the self-paced trials with the dominant (right) hand, a significantly reduced RATE was found in the MS group with respect to the HC group in both spontaneous and maximal velocity conditions (SV: 1.93±0.09 Hz vs. 2.40±0.05 Hz, p<0.0001; MV: 2.67±0.10 Hz vs. 3.25±0.06 Hz, p<0.0001).

The analysis of the metronome-paced motor sequence with the right hand (2 Hz) showed significant differences in motor performance between the MS and the HC groups. TD significantly increased (246.67±10.30 ms vs. 213.82±4.68 ms, p = 0.003) whilst ITI slightly decreased (269.24±10.92 ms vs. 279.63±5.10 ms, p = 0.049) in the MS group with respect to the HC group.

In the bimanual condition (2 Hz_bim), we found a significantly higher IHI in the MS group with respect to the HC group (41.92±4.48 ms vs. 18.87±0.87 ms, p<0.0001), indicating impairment in bimanual coordination in patients with MS.

### Parameters better describing the patient's finger motor impairment

We included all the motor performance parameters significantly different between the HC and MS groups (RATE in both SV and MV conditions, TD, ITI and IHI) in a multivariate model with group (HC and MS) as dependent variable. Two parameters were found to be independently able to discriminate the MS group from the HC group: RATE (SV) (odd ratio (OR) = 0.17, 95% confidence interval (CI) = 0.05–0.58, p = 0.005) and IHI (2 Hz_bim) (log-transformed, OR = 23.17, 95%CI = 6.26–85.74: p<0.001). The discriminating ability of the model including these two motor performance parameters was high, as assessed by the ROC analysis (Figure? 3) (AUC = 0.89 (95%CI = 0.82–0.96, p<0.001)), even when evaluated using a cross-validation procedure based on the leave-one-out method (AUC = 0.88 (95%CI = 0.81–0.95, p<0.001)).

**Figure 3:**
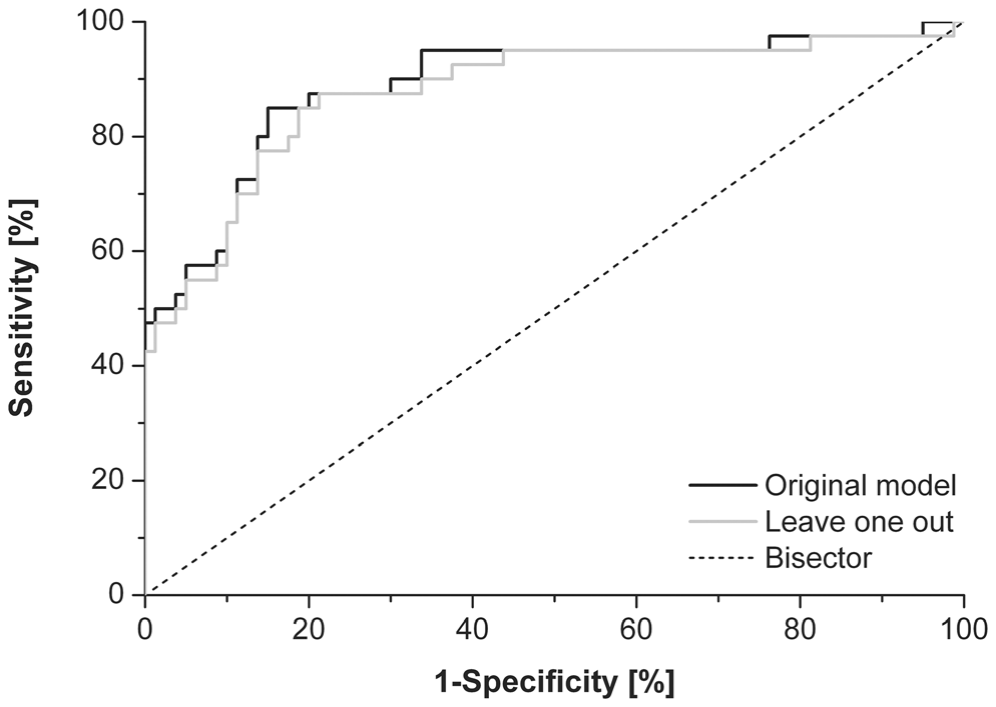
ROC analysis. The ability of the model of discriminating patients with MS from the HC group is demonstrated by the area under the ROC curve (AUC = 0.89 (95%CI = 0.820.96, p<0.001)), even when evaluated using a cross-validation procedure based on the leave-one-out method (AUC = 0.88 (95%CI = 0.81–0.95, p<0.001)).

### Relationship between finger motor performance and clinical evaluation

The correlations of each motor performance parameter with clinical variables are shown in Table? 2.

**Table 2:** Correlations between the motor performance parameters and the clinical scores in the MS group, with age as control variable (correlation coefficient r values and p values are reported).

	EDSS		MSFC		9-HPT		T25W		PASAT		MFIS		MFIS physical		MFIS cognitive		MFIS psychosocial	
Parameter (condition) [unit]	r	p	r	p	r	p	r	p	r	p	r	p	r	p	r	p	r	p
RATE (SV) [Hz]	−0.11	0.50	0.11	0.51	−0.23	0.15	−0.02	0.90	0.02	0.92	−0.18	0.26	−0.30	0.06	−0.02	0.91	−0.20	0.21
RATE (MV) [Hz]	−0.39	0.01*	0.47	0.002*	−0.45	0.004*	−0.18	0.28	0.31	0.05	−0.21	0.20	−0.38	0.02*	−0.07	0.66	−0.14	0.40
TD (2 Hz) [ms]	0.09	0.59	−0.40	0.01*	0.45	0.004*	0.03	0.87	−0.25	0.12	−0.14	0.40	−0.08	0.64	−0.03	0.86	−0.09	0.58
ITI (2 Hz) [ms]	0.09	0.59	0.23	0.16	−0.37	0.02*	0.15	0.35	0.16	0.32	0.27	0.09	0.23	0.17	0.12	0.45	0.23	0.16
IHI (2 Hz_bim) [ms]	0.56	<0.0001*	−0.40	0.01*	0.24	0.14	0.30	0.07	−0.38	0.02*	0.05	0.77	0.20	0.22	−0.11	0.50	0.01	0.93

* indicates statistical significance.

SV = Spontaneous Velocity condition. MV = Maximal Velocity condition. 2 Hz = metronome condition (tone set at a rate of 2 Hz). 2 Hz_bim = metronome condition with both hands simultaneously. RATE = movement speed. TD = Touch Duration. ITI = Inter Tapping Interval. IHI = Inter Hand Interval. EDSS = Expanded Disability Status Scale. MSFC = Multiple Sclerosis Functional Composite. 9-HPT = 9-Hole Peg Test. T25W = Timed 25-Foot Walk. PASAT = Paced Auditory Serial Addition. MFIS = Modified Fatigue Impact Scale.

In particular, RATE (MV) significantly correlated with EDSS (r = −0.39; p = 0.01), MSFC (r = 0.47; p = 0.002), 9-HPT (r = −0.45; p = 0.004) and MFIS-physical subscore (r = −0.38; p = 0.02). Further, significant correlations were found between IHI and EDSS (r = 0.56; p<0.0001), MSFC (r = −0.40; p = 0.01), Paced Auditory Serial Addition (PASAT) (r = −0.38; p = 0.02). No motor performance parameter correlated with Timed 25-Foot Walk (T25W).

In addition, we defined a “Finger Motor Impairment score” based on the logistic model resulting from the multivariate analysis and reported it according to different levels of disability in Figure? 4: an increase in finger motor impairment as measured by the glove system was found with increasing disability, passing from HC subjects, to MS patients with EDSS = 0, patients with EDSS = 1−2, patients with EDSS = 2.5−4 and patients with EDSS>4 (p for trend <0.001). Interestingly, a difference with respect to the HC group was already evident in the MS patients with EDSS = 0 (p = 0.049) and became highly significant starting from the group of MS patients with EDSS 1−2 (p<0.001).

**Figure 4:**
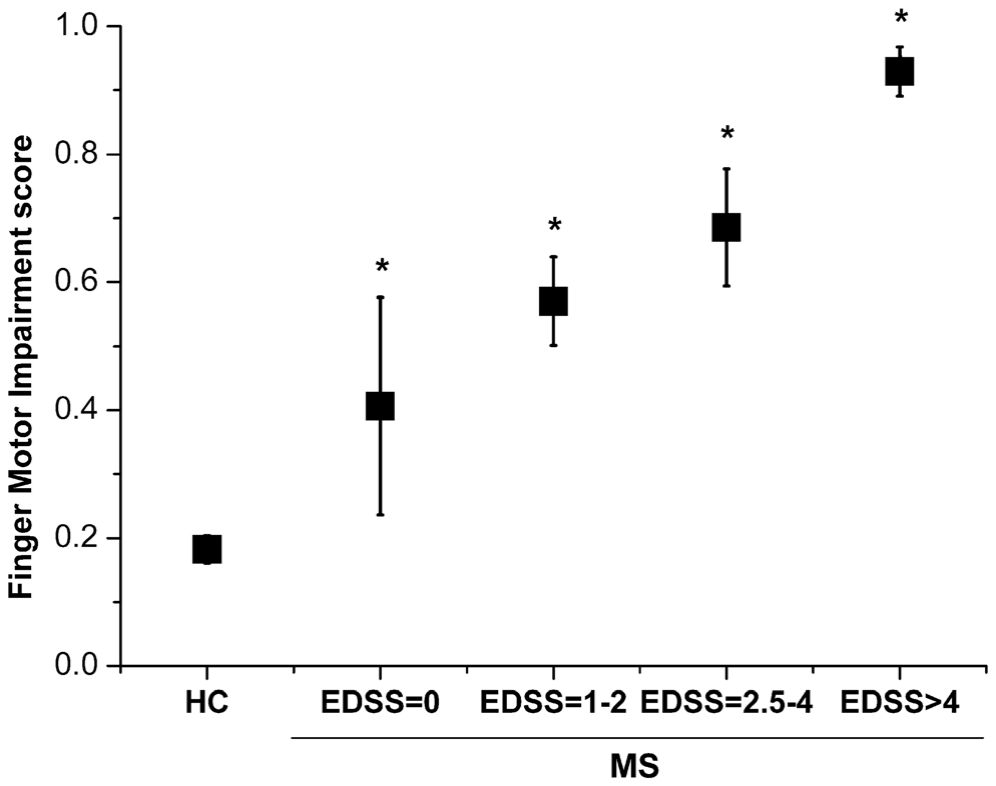
Relationship between “Finger Motor Impairment score” and EDSS. The weighted combination of motor performance parameters (“Finger Motor Impairment score”) given by the multivariate model was able to discern subjects from no disability to high level of disability (HC; patients with MS: EDSS = 0, EDSS = 1−2, EDSS = 2.5−4, EDSS>4) (p for trend <0.001). * indicates statistical significance with respect to healthy controls (HC group). Error bars indicate standard error of the mean.

## Discussion

In this work we quantitatively evaluated uni and bi-manual motor performance in a cohort of patients with MS and in a group of age- and gender – matched healthy controls.

First, we found that in healthy subjects there was a good repeatability of most of the finger motor performance parameters evaluated in single trials. Indeed, the behavioral protocol proposed to the two groups (HC and MS groups) included only single trials (one per condition) because a simple protocol could ensure a high patient's compliance. However, it should be considered that above all in longitudinal studies it could be useful to repeat each condition twice, to increase the reproducibility of the parameters assessment (e.g., the single ICC of ITI was 0.39 but it became 0.55 when averaged on two trials).

Then, a multivariate model revealed that two finger motor performance parameters (RATE (SV) and IHI (2 Hz_bim)) were able to independently distinguish the two groups, indicating that these two parameters taken together better discriminated than each one separately. The logistic regression analysis showed that a weighted combination of the two parameters revealed by the multivariate model (RATE (SV) and IHI (2 Hz_bim)) was able to discriminate HC from MS patients with very low EDSS scores: a significant difference was already evident for patients with EDSS = 0. This finding demonstrates a high sensitivity of the motor performance parameters in detecting a subclinical impairment, not revealed by the EDSS.

Considering the two parameters separately, we found that movement rate (RATE) was significantly reduced in patients with MS in both the spontaneous and maximal velocity condition. This finding is in agreement with previous reports showing a general motor slowing in patients with MS [9], [15], [16], [17], [18]. Further, when investigating bimanual coordination by means of the inter hand interval (IHI) we found that patients with MS had an impairment in the coordination of bimanual finger movements since they showed larger IHI values than HC. Similarly, in the literature, patients with MS were found to be slower than normal subjects when performed the Bimanual Coordination Test. Precisely, a deficit in bimanual motor coordination was observed only in those patients with abnormal cross-callosal evoked potentials, evidence of inefficient callosal transmission, supporting the conclusion that deficits in bimanual motor coordination occur in MS and are related to callosal dysfunction [17]. This topic was expressly evaluated in a previous study [9], showing that the degree of damage in the anterior callosal portions mainly influences bimanual coordination and, in particular, the movement phase preceding the finger touch. Interestingly, IHI significantly correlated with PASAT. In fact, as in PASAT, during bimanual coordination the integrity of attentional and coordination processes is necessary to successfully accomplish the task. More specifically, the PASAT and its visual analogue, the Paced Visual Serial Addition Test (PVSAT), have been widely used to test sustained attention, working memory and speed of information processing [19], [20]. Functional MRI (fMRI) studies based on these tests showed activations of fronto-parietal brain areas involved in attention processing [21], [22], [23].

Nevertheless, it should be underlined that all the glove parameters were significantly different between the two groups. In particular, when right-hand finger movements were metronome-paced at 2 Hz, patients with MS showed a significantly larger TD. This finding might indicate that patients with MS need more time to evaluate the finger contact, recognize the finger that has been actually touched and then identify the next finger to be touched before moving forward in the performance of the correct sequence, suggesting a sensorimotor integration impairment in these patients.

In general, the correlation coefficients obtained evaluating the relationships of the glove parameters with clinical variables, were found to be low, as a priori expected. This finding could indeed represent a point of strength of the proposed methodology; in fact it is a confirmation that the glove parameters are able to assess domains that are not fully accounted for by the EDSS and not accounted at all for example by the T25W test. Then, it could be suggested that our method potentially provides new parameters measuring different aspects of impairment and they are not thought as a substitute of the EDSS, but as an addition to the patient evaluation.

Further studies are required to optimize and validate the obtained score indicating finger motor impairment in an independent set of data, in order to define the optimal combination of glove parameters discriminating patients and controls.

Finally, the assessment of finger motor impairment in MS could re-balance the motor disability scale, now heavily weighted toward ambulation, enhancing upper limb evaluation together with the MSFC, and provide an affordable tool to be utilized in new clinical studies to assess the impact of MS therapies, also in the early phases of MS.

It is also worth noting that finger impairment and manual ability are not closely related in a predictable straightforward relationship [24]. Therefore, to further evaluate the construct validity of this methodology, future studies should also include specific scales measuring the hand ability level (i.e., the capacity to manage daily activities requiring the use of the upper limbs whatever strategies involved), such as the ABILHAND questionnaire, recently built and calibrated for patients with MS [25].

## Conclusions

In conclusion, we demonstrate that assessing a score indicating finger motor impairments allows discriminating healthy controls and MS patients, even with very low disability. If confirmed in larger studies, this sensitivity could be of crucial importance for monitoring the disease course and the treatment effects in early MS patients, when changes in the EDSS are small or absent.
